# Comprehensive review of multiomics applications and remediation of plant heavy metal toxicity

**DOI:** 10.1007/s44154-025-00233-w

**Published:** 2025-09-16

**Authors:** Tamana Khan, Labiba Shah, Sabba Khan, Owais Ali Wani, Zahid Nabi Sheikh, Baseerat Afroza, Rizwan Rashid, Faheem Shahzad Baloch, Sheikh Mansoor

**Affiliations:** 1 Division of Vegetable Science, Faculty of Horticulture, Sher e Kashmir University of Agricultural Sciences and Technology of Kashmir, Shalimar- 190025, Jammu and Kashmir, India; 2 Division of Soil Science and Agricultural Chemistry, Faculty of Agriculture, SKUAST Kashmir, Srinagar, 193201 India; 3 Division of Biochemistry, Kashmir University of Agricultural Sciences and Technology of Jammu, Shere , Jammu and Kashmir, 180009 India; 4 Department of Plant Resources and Environment, Jeju National University, Jeju, 63243 Republic of Korea; 5 Department of Biotechnology, Faculty of Science, Mersin University, Mersin33343, Türkiye; 6 Department of Horticulture, Faculty of Agriculture, Recep Tayyip Erdoğan University, Rize, 53300 Türkiye

**Keywords:** Heavy metals, Plant toxicity, Stress signalling, Multiomics, Remediation

## Abstract

Heavy metal pollution severely impacts plant health by inhibiting growth, photosynthesis, enzyme activities, and causing oxidative stress. Plants respond to such stress by activating complex defense mechanisms involving reactive oxygen species and different signaling pathways. These pathways are pivotal in triggering plant defense responses and are currently a major focus of research. Understanding the complex mechanisms of heavy metal uptake, transport, chelation, and signaling can guide strategies to improve plant resilience and stress tolerance. In this review, we aim to highlight the key heavy metals found in soil and the environment, along with their mechanisms of accumulation in plants. We also explore the defense responses of plants through various signaling pathways such as calcium (Ca^2+^), MAP kinase, and hormone signaling. Additionally, we emphasize the importance of understanding advanced omics technologies, including transcriptomics, metabolomics, and bioinformatic tools, in enhancing our knowledge of plant resilience and stress tolerance.

## Highlights

• Heavy metals are major environmental concern with consequences on plant and public health.

• Heavy metal pollution has increased globally decreasing agricultural growth and productivity.

• Calcium functions as a messenger in both the regular physiology and against various stressors.

• Phytohormones have antagonistic and synergistic effects that control response to stressors.

• Bioinformatic tools aid in processing, analyzing, and interpreting data in stress research.

## Introduction

Heavy metals, defined as elements with densities at least five times greater than water, pose significant health risks to both humans and plants. Key heavy metals of concern include chromium (Cr), arsenic (As), iron (Fe), lead (Pb), zinc (Zn), copper (Cu), cadmium (Cd), and nickel (Ni) (Mansoor et al. 2023; Ohiagu et al. 2022). Major sources of human exposure to these metals arise from agriculture, mining, industrial activities, and improper waste disposal (Tchounwou et al. 2012; Alengebawy et al. 2021). The mechanisms underlying heavy metal toxicity are complex; however, one common mechanism involves the induction of oxidative stress. Heavy metals can stimulate the production of reactive oxygen species (ROS), inhibit enzyme functions, and disrupt antioxidant defenses (Mansoor et al. 2022; Zhuang et al. 2009). In humans, heavy metal toxicity can interfere with DNA and nuclear proteins, leading to structural and functional damage that may initiate processes such as carcinogenesis, apoptosis, and dysregulation of the cell cycle (Engwa et al. 2019; Tchounwou et al. 2012).

In agricultural contexts, the accumulation of heavy metals in soils presents a significant concern due to its potential impact on food systems, crop productivity, and the health of soil organisms (Pahlsson 1989; Nagajyoti et al. 2010). The detrimental effects of heavy metals on plant metabolism, growth, and biomass production highlight the urgency of addressing pollution in contaminated areas. (Manara 2012; Mansoor et al. 2023). While certain metals are essential micronutrients for plant metabolism—such as copper (Cu), manganese (Mn), cobalt (Co) and zinc (Zn)—elevated levels can be toxic. Foy et al. (1978) reviewed the physiological mechanisms underlying heavy metal toxicity in plants. Excessive concentrations of heavy metals can induce stress symptoms in plants; however, many species have developed adaptive systems to mitigate these effects. Plants employ strategies such as metal chelation, transport, compartmentalization, and the synthesis of chelators and proteins that bind to metals. Chelation is a common intracellular detoxification process regulated by thiol molecules such as glutathione (GSH), phytochelatins (PCs), and metallothioneins (MTs). Elevated metal levels lead to oxidative stress by generating reactive oxygen species (ROS). (Sharma et al. 2012). GSH, found in various parts of plant cells, is crucial for synthesizing phytochelatins that facilitate metal detoxification. Additionally, the regulation of ROS levels by the ascorbate–glutathione (AsA–GSH) pathway enhances plant resistance to metal-induced oxidative stress (Hasan et al. 2017). These systems are essential for helping plants withstand metal and metalloid stress.

Globally, there is increasing concern regarding the harmful effects of heavy metal poisoning on ecosystems and human health (Duruibe et al. 2007; Zhang et al. 2017a, b). Soil, water, and agricultural products near industrial facilities—such as zinc smelting plants—often exhibit elevated concentrations of heavy metal pollutants. The consequences for local ecosystems and communities exposed to high levels of copper (Cu), zinc (Zn), cadmium (Cd), and lead (Pb) extend to livestock and human populations alike (Duruibe et al. 2007; Reis et al. 2010; Briffa et al. 2020). Shen et al. (2019) documented significant differences between polluted and control sites; soil, irrigation water, forages, and food grown in impacted areas contained markedly higher concentrations of heavy metals. The effects are not limited to environmental degradation but also manifest as hazardous accumulations in human biological samples and animal tissues. Furthermore, exposure to heavy metals can cause anemia in humans and animals, marked by hypochromic and microcytic characteristics (Duruibe et al. 2007; Reis et al. 2010). Decreases in protein characteristics and serum total antioxidant capacity further underscore the systemic effects of heavy metal pollution on physiological processes (Shen et al. 2019). Given these serious findings, prompt action and cleanup operations are necessary to mitigate health risks for humans and livestock residing near sources of industrial pollution.

**Fig. 1:**
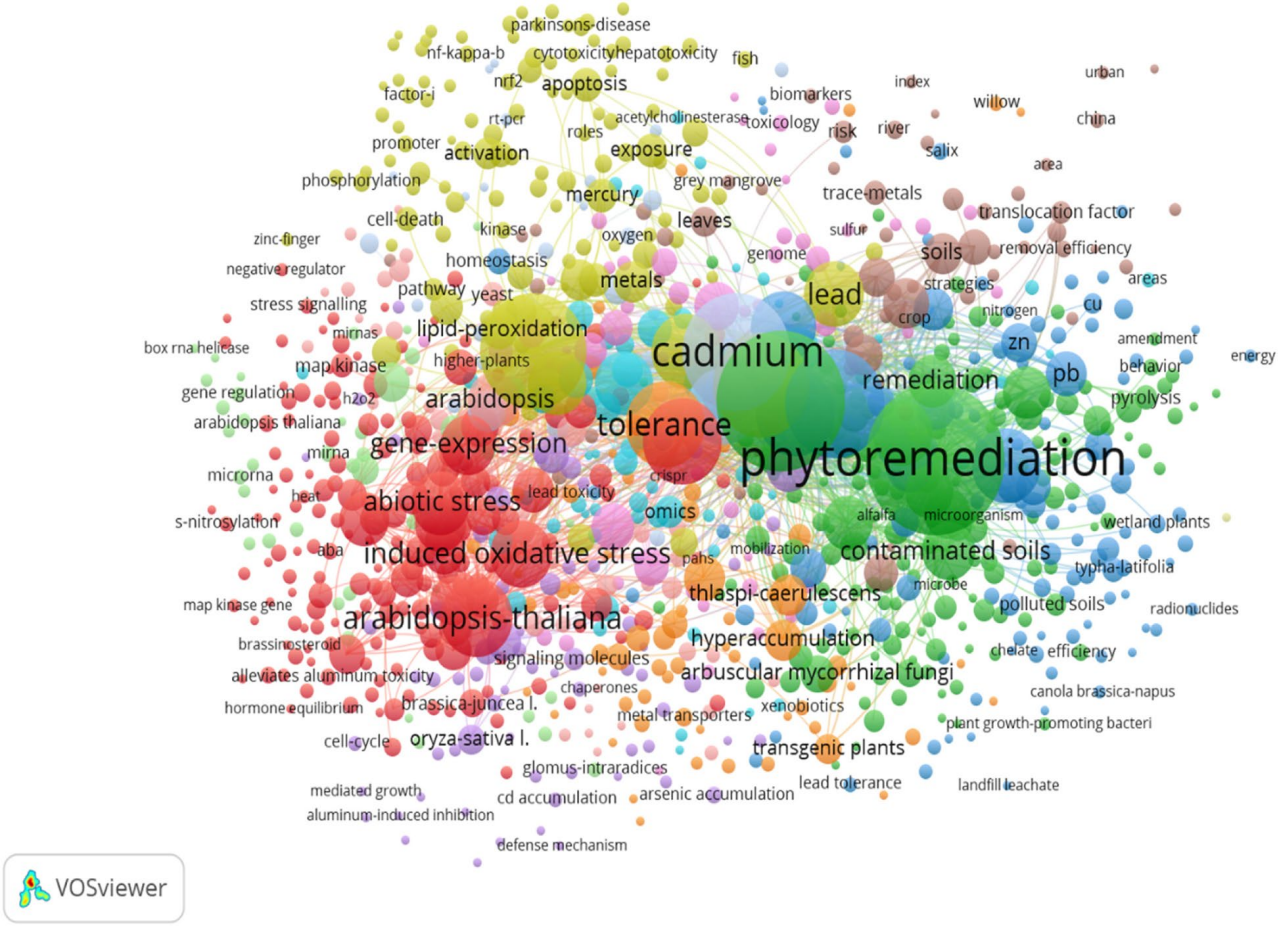
In the last decade, a hotspot analysis was conducted on literature focusing on the intersection of “omics in heavy metal stress, remediation, pyrolysis, and heavy metal stress signaling”. Each point in the analysis represents the weight of keywords in the literature, with larger points indicating greater focus on those keywords. Coupling relationships between points are indicated by lines connecting them

To better understand these issues, we conducted a literature survey using key terms in VOS Viewer to construct bibliometric networks on omics related to heavy metal stress, signaling pathways, and remediation efforts. These networks connect publications through citations and keywords to reveal functional relationships. VOS Viewer aids in visualizing co-occurrence networks by extracting significant terms from scientific literature (Fig. 1). This review addresses various aspects of heavy metal toxicity in plants, stress signaling mechanisms, and multi-omics applications for exploring adaptations to heavy metal stress. It also examines strategies for protecting affected populations and ecosystems through physical, biological, and biotechnological methods aimed at mitigating heavy metal toxicity in plants.

## Most common heavy metals and their impact on plants

Plants due to their stationary nature, experience environmental disturbances throughout their life cycle. Heavy metals, in particular, are getting a lot of attention in recent years as a major stressor for plants (Rai et al. 2016). These heavy metals, coming from transition elements like lanthanides, actinides, and metalloids, can be quite dangerous even at very low levels (Gonzalez et al. 2021). The heavy metals (HMs), such as arsenic (As), cadmium (Cd), lead (Pb), chromium (Cr)and mercury (Hg), are not vital to plant metabolism and can significantly lower agricultural productivity once concentrations of these elements reach supra-optimal levels (Bhat et al. 2019). Plants experience redox imbalance and increased levels of lipid peroxidation as a result of heavy metal toxicity, which causes an excessive production of reactive oxygen species (ROS) (Branco-Neves et al. 2017).

### Lead (Pb)

Pb is a non-biodegradable heavy metalthat poses a significant threat to environmental health, with plants being at the forefront of exposure. Industrial activities, mining, and Pb-based paints contribute to Pb contamination in soil and water, leading to its uptake by plants (Kumar et al. 2020). Pb disrupts vital physiological processes, hinders growth, and can even enter the food chain. Plants absorb Pb primarily through their roots. The extent of uptake depends on various factors, including soil properties (pH, organic matter content), Pb speciation (chemical form), and the specific plant species. Plants employ different mechanisms to regulate Pb uptake. Pb is absorbed passively as Pb^2^^+^, a divalent ion, on the surface of root hairs (Yang et al. 2004). In comparison to the other metals, 95% of Pb is better confined to the root hair because it is both firmly bonded to the organic materials in the soil and less soluble at pH values higher than 5, and only 5% is translocated to the aerial tissues. Pb uptake, which is driven by the transpiration process, occurs via xylem tissues through apoplast motion and is obstructed by Casparian strips of endodermis (Zulfikar et al. 2019). After that, with the help of vascular flow and symplast movement, it is translocated and sequestered to the aerial sections. Higher quantities of Pb cause major harm to plants by interfering with the uptake of vital mineral nutrients (Ca^2^^+^, Fe^2^^+^, Mg^2^^+^, Mn^2^^+^, Zn^2^^+^, and K^+^). Exposure to Pb can cause plants to experience oxidative stress, which can result in altered genomic DNA, decreased seed germination, and decreased seedling development (Jamlaet al. 2021). Accelerated suppression of root growth, stunted plant growth, blackening of the root system, and chlorosis are the outward signs of Pb poisoning. Pb reduces the activity of enzymes, water balance, mineral nutrition, and photosynthesis (Sharma et al*.* 2005). Pb is considered to be one of the most effective metal ions for membrane degradation and for inhibiting the function of chloroplastic ATP synthetase/ATPase. Furthermore, excessive reactive oxygen species accumulation by Pb can induce lipid peroxidation, enzyme inactivation, and protein oxidation in plants (Bhardwaj et al. 2022).

### Mercury (Hg)

Quicksilver, or Hg, is the third most hazardous metal according to the Agency for Toxic Substances and Disease Registry (ATSDR List, 2017). Hg can be found in nature in three different forms: elemental (Hg), inorganic (Hg^+^, Hg^2+^), and organic (MeHg). It exists in soil as HgS (cinnabar), a less hazardous water-insoluble form. The predominant species in the environment is Hg^2+^ (Beckers et al. 2017). Under anaerobic conditions, the methylation process transforms inorganic Hg into toxic (monomethyl, RHg^+^, and dimethyl, R_2_Hg) organic Hg (Hong et al. 2012). The primary determinants of Hg uptake by plants are species type, Hg speciation, and varied soil characteristics. The two bioavailable forms of Hg in soil are Hg^2+^ and Me-Hg, with Me-Hg being more harmful and fatter soluble (Khanam et al. 2020). Because Hg is hydrophilic by nature, it is readily absorbed by the roots, translocated to the shoots, and then expelled as a gas back into the atmosphere. But it's widely recognized that, in plants, Hg tends to accumulate mainly in the roots instead of moving to the shoots (Xu et al. 2015). Plants under stress can exhibit various signs of Hg phytotoxicity. For example, it impedes the flow of water, breaks down macromolecules, interferes with the metabolism of organelles, and ultimately leads to oxidative stress (Ghori et al. 2019). Hg also induces the breakdown of photosystem activity because it has a higher binding energy than Mg for attaching to the chlorophyll Pheo ligand (Bechaieb et al. 2016). Many plant species are known to experience oxidative stress when subjected to excessive Hg (Singh et al. 2016a, b).

### Arsenic (As)

As is a non-essential HM that is regarded as a non-threshold Class 1 carcinogen. Its bioavailability to the soil ecosystem is increasing due to rapid anthropogenic activities and natural processes (Wu et al. 2021) As can be found in nature in the chemical forms of arsenite (As^3+^), arsenate (As^5+^) and mono- and di-methylarsinic acid (MMA, DMA), the most harmful of which is inorganic As^3+^. As^5+^ is the predominant species in aerobic conditions, whereas As^3+^ predominates in anaerobic soil (Srivastava et al. 2017).As it approaches the rhizosphere zone, it works to assimilate nutrients through assimilatory routes before moving them to the grains by various transporters. As^5+^ is absorbed into the roots by phosphate transporters because it resembles inorganic phosphate (Pi) found in soil. Pi/As^5+^ affinity varies between and across the various plant types (DiTusa et al. 2016). Membrane channel proteins called aquaporins (AQPs) are another class of transporters that can absorb As^3+^ species in anaerobic field circumstances together with other vital mineral nutrients. As^3+^ uptake in a variety of plants has been described for the AQPs family subclasses, which include tonoplast core proteins, plasma membrane core proteins, and nodulin-like intrinsic proteins (Castrillo et al. 2013). Plants are affected by arsenic in both morphological and physiological ways. Common signs of arsenic stress in plants include increased activity of glutathione reductase (GR), ascorbate peroxidase (APX), superoxide dismutase (SOD), and lipid peroxidation (Beniwal et al. 2023). Phosphate transporters are further activated by arsenic exposure and are accountable for Phosphate uptake as well (Sun et al.^2018^). Other essential functions like N_2_ fixation, photorespiration, sulfur metabolism, and metabolic pathways are also inhibited by arsenic stress, which results in plant death and growth retardation (Franic et al. 2019).

### Chromium (Cr)

Many chemical forms of Cr are present in soil, the most common being chromate (Cr^4+^) and chromite (Cr^3+^), which both have remarkably different biogeochemical characteristics (Kimbrough et al. 1999). Owing to the intricate electrical chemistry of Cr, it has proven to be very challenging to identify the mechanism driving plant toxicity (Shrivastava et al. 2002). As a hazardous and non-essential element for plants, no transporters or channels unique to Cr have been found in plants as of yet. Rather, Cr is transported by certain critical element transporters in most plant species. Many physiological, morphological, and metabolic characteristics of plants are negatively impacted by Cr exposure, which eventually results in plant mortality. Cr exposure changes antioxidant activity, produces more reactive oxygen species (ROS), and impacts photosynthesis, nutrient uptake, and plant development. Because Cr toxicity affects the majority of agriculturally significant crops, including pulses, cereals, vegetables, etc., it has a significant effect on our sustainable agriculture and food security (Hayat et al. 2012). The overall harmful effects of Cr are determined by its metal evolution, which varies significantly in plants in terms of the rate of uptake, accumulation, and translocation. The carriers of necessary anions, such as sulfate, are involved in the active mechanism of Cr^4+^transport. During transportation, it has been noted that Cr competes with sulfur (S), phosphorous (P), and iron (Fe). Because phosphate and sulfate share structural similarities with Cr^4+^, plants typically absorb Cr^4+^ through phosphate or sulfate transporters (De Oliveira et al. 2014). Plant root growth seemed highly inhibited by chromium stress. The suppression in proliferation of cells and corresponding decrease in cell size is promoted by Cr (Peralta et al. 2001). Cr hinders roots from proliferating into new cells and decreases the length of roots, which reduces their capacity to take up water and nutrients and impairs the growth of shoots. After absorption, it has been found that the primary pathway for Cr to be transported is through the plant xylem (Ali et al. 2023).

### Cadmium (Cd)

After Hg and Pb, Cd is classified as a Group I human carcinogen and is the third most dangerous contaminant as ranked by US Environmental Protection Agency (EPA) (Jaishankar et al. 2014). Because Cd is present in the environment, soil, and water, it can easily bioaccumulate in the food chain. Cd contamination of soil is caused by industrial effluents, phosphate fertilizer application, sewage disposal, smelting, mining, and farming runoff. The soil's pH, mineral composition, water content, plant species, and the proportion of nutritional minerals in the surrounding environment all affect the bioavailability of Cd (Verbruggen et al.^2009^). Cd is more soluble and accessible for plant absorption at acidic pH values. In the environment, Cd can be found either in the ionic form (Cd^2+^) or bonded as chelates (Clemens et al. 2016). Through transpiration flow and non-specific HM transporters, root cell walls absorb Cd^2+^. Apoplastic and symplastic pathways are the two primary routes via which Cd is transported in plants. The symplastic route is employed when Cd binds to metal transporters (Song et al. 2017). The plant can absorb Cd more easily through the apoplast pathway. The root surface absorbs Cd during H^+^ exchange subsequent to the breakdown of carbonic acid (Moravčíková et al. 2023). Cd causes oxidative stress and impedes food intake, which hinders plant growth, chlorophyll pigments, seed germination, water relations, and vital physiological functions (Hafeez et al. 2023). Chlorosis, leaf rolling and stomatal closure are signs of Cd stress. Chlorophyll and carotenoid levels are reduced by Cd, whichadversely affect both photosystems and the light-harvesting complex II (LHCII) (Clemens et al. 2006). Table 1 provides a brief summary of the harmful effects of heavy metals on plants.

**Table 1:** Toxic effects of heavy metals on plant growth and development

Heavy Metal	Impacts on Plants	References
**Lead**	In spinach and kale plants, reduced levels of chlorophyll and altered root structure	Lestari et al. 2022
	In maize reduced plant biomassand decline in the germination percentage	Hussain et al. 2013
	Inhibition of CO_2_ fixing enzymes in Oats	Aslam et al. 2021
**Mercury**	Inhibits tiller and panicle formation in rice	Kibra 2008
	Chlorosis in tomato	Shekar et al. 2011
	Oxidative stress and interferes with metabolic pathways in leafy vegetables	Das et al. 2022
**Arsenic**	In soybean plants, altered levels of jasmonic acid and abscisic acid	Vezza et al. 2021
	Alteredradical development of radish and lettuce seeds	Grafe. 2000
	Unfavourable growth changes in onion	Sushant et al. 2010
**Chromium**	Diminished germination and growth in maize	Garba et al. 2021
	Abnormal stomatal conductivity, and decreased plant growth	Rath and Das 2021
	Chromium increases MDA lipid contents in wheat	Ali et al. 2015
**Cadmium**	In wheat and sunflower roots, diminish ATPase activity of the plasma membrane	Fodoret al. 1995
	In garlic, inhibited shoot growth	Jiang et al. 2001
	Cadmium inhibits root growth and shoot growth in maize	Wang et al. 2007

### Aluminium (Al)

One of the main components of soil is aluminium, which dissolves in the soil in a variety of ionic forms, the most hazardous of which is Al^3+^. Al has the ability to produce several hydroxy-Al and polynuclear species in solution in addition to the Al^3+^cation. It has been discovered that high concentration of Alreduces germination, root elongation, plant development, and chlorophyll synthesis. (Ali and Gill 2022). Although there are several impacts of Al toxicity on crops, it is usually the root tip that is the primary site of Al toxicity (Wang et al. 2020). The plant's root system is the area most readily impacted by Al poisoning. Al phytoxicity inhibits the cell division process which causes the root apices to swell and get damaged, the root to become stunted and brittle, and the root hair to develop poorly. It was reported that Al toxicity results in severe root damage that impairs water and ion absorption. It interacts with lipids to cause lipid peroxidation, which changes the way the plasma membrane functions (Barcelo and Poschenrieder. 2002). Al stress led to damage to the plasma membrane in peanuts by causing the peroxidation of lipids in the root tip cell membrane. Following the damage, cytochrome C was released into the cytoplasm of the injured plasma membrane, causing an excess of Ca^2+^ to be present. This caused the root tip cells to undergo programmed death, which in turn stopped the growth of the roots (Yao et al. 2016). It was reported that Al causes callose to accumulate in the plasmodesmata of wheat root cells, preventing cell-to-cell transport (Sivaguru and Horst. 1998).In two recent studies, it was demonstrated that Al caused a decrease in both the photosynthetic rate and chlorophyll concentration (Ali et al. 2008) Tables 2 and 3.

**Table 2:** Summary of the sources, toxic effects, and specific plant responses of different heavy metals

Heavy Metal	Sources	Toxic Effects	Plant Responses
Lead (Pb)	Industrial activities, mining, lead-based paints	Disrupts nutrient uptake, causes oxidative stress, hinders growth, and damages chloroplasts and DNA	Chlorosis, stunted growth, blackened roots, reduced enzyme activity, and inhibited photosynthesis
Mercury (Hg)	Industrial emissions, methylation of Hg in anaerobic soils	Impedes water flow, disrupts organelle metabolism, induces oxidative stress, and affects photosystem activity	Root accumulation, reduced photosynthesis, membrane damage, and lipid peroxidation
Arsenic (As)	Anthropogenic activities, fertilizers, mining	Affects N2 fixation, photorespiration, and sulfur metabolism; induces oxidative stress and inhibits growth	Increased antioxidant activity (SOD, APX), growth retardation, and metabolic inhibition
Chromium (Cr)	Industrial waste, mining, and electroplating	Generates ROS, alters photosynthesis, disrupts nutrient uptake, and inhibits cell division and elongation	Shortened root length, reduced water and nutrient uptake, stunted shoots, and impaired growth
Cadmium (Cd)	Industrial effluents, fertilizers, sewage, mining	Causes oxidative stress, hinders photosynthesis, reduces seed germination, and affects water and nutrient uptake	Chlorosis, leaf rolling, stomatal closure, reduced chlorophyll content, and impaired light-harvesting complexes
Aluminum (Al)	Acidic soils, naturally occurring in soil as Al3 +	Inhibits root elongation, causes lipid peroxidation, and induces ROS production	Swollen root tips, brittle roots, reduced water absorption, callose accumulation, and DNA damage
Nickel (Ni)	Natural processes, industrial emissions, fertilizers	Impairs nutrient absorption, reduces seed germination, and causes chlorosis and necrosis	Slower seedling growth, reduced photosynthesis, nutrient deficiencies, and ROS production
Zinc (Zn)	Mining, smelting, fertilizers, industrial processes	Disrupts root architecture, hinders photosynthesis, and induces oxidative stress	Shortened primary roots, reduced PSII efficiency, leaf chlorosis, and impaired aerobic respiration

**Table 3:** Mitigation of heavy metal toxicity in plants

S.No	Heavy metal toxicity	Mitigation strategy	References
	**Phytoremediation**		
1	Cd	*Azolla pinnata*, *Eleocharis acicularis*, *Rorippa globosa*, *Solanum* *photeinocarpum*	Rai 2008
2	Cr	*Pteris vittata*	de Oliveira et al. 2014
3	Pb	*Euphorbia cheiradenia*	Malayeri et al. 2013
4	Ni	*Alyssum bertolonii*, *Alyssum caricum*, *Alyssum corsicum*, *Alyssum murale*	Bani et al. 2010
5	As	*Corrigiola* *telephiifolia*	García-Salgado et al. 2012
6	Zn, Cu	*Eleocharis acicularis*	Ha et al. 2009

It was also observed that in pea and tobacco, ATP was depleted and reactive oxygen species were produced at later stages (Panda et al. 2008). Al toxicity has also been reported to increase ROS and DNA damage, inhibit the synthesis of ATP and interfere with transcriptome sequencing (Ali and Gill 2022).

### Nickel (Ni)

Natural processes such as wind, weathering of rocks, and wildfires release Ni into the atmosphere. Ni is thought to be a necessary element for plant growth and development, but only in small amounts (0.05–10 mg/kg dry weight), as large concentrations prove harmful to plants (Bhalerao et al. 2015). Firstly, Ni toxicity has an impact on seed germination. Ni toxicity reduces metabolic processes, cell wall flexibility, and cell division, which has detrimental effects on seed germination and seedling growth. Ni toxicity has been shown to significantly reduce seed germination (Zhang et al. 2007). Furthermore, inhibited cell division is linked to Ni-induced inhibition of cell growth (Bhalerao et al. 2015). According to Rao and Sresty (2000) in pigeon pea seed germination decreased by 20% when exposed to Ni toxicity as opposed to a control (no Ni stress). Influence of Ni toxicity on maize seedlings was studied and a slower seedling development was found when Ni toxicity was higher (2.0 mM)(Bhardwaj et al. 2007). Ni poisoning prevents nutrients from being taken up and absorbed by the plant, which causes nutrient deficiencies in plants (Ahmad et al. 2007). Because of their chemical affinity for Fe, Zn, and Mg, organic macromolecules that are bound by high concentrations of Ni get denatured and disrupted, making it easier for those elements to replace(Wang et al. 2015). Ni induces necrosis and chlorosis in plants by interfering with their ability to absorb and metabolise iron. Ni toxicity has been found to cause reduction in grain yield in wheat due to nutrient deficit (Wang et al. 2015). In a comparable way, in wheat magnesium deficiency under Ni stress causes chlorophyll to deteriorate, which in turn caused necrosis and chlorosis in leaves (Shukla and Gopal 2009).Also, in potato magnesium deficiency under Ni stress causes chlorophyll to deteriorate, which in turn causes necrosis and chlorosis in leaves (Shukla and Gopal 2009). In guava plant development and leaf gas exchange gradually declined and significant K^+^ losses occurred due to Ni toxicity (Bazihizinaet al. 2015). Presence of reactive oxygen species (ROS) such as superoxide (O_2_^•^^–^), hydroxyl radical (OH^•^), hydrogen peroxide (H_2_O_2_), and alkoxy radicals in plants was reported when there was an excess of Ni concentration in the growing media was also reported (Gajewska and Skłodowska 2007).

### Zinc (Zn)

Zn naturally occurs in soil as a result of mother rocks' leaching during pedogenetic processes. Apart from natural phenomena, industrial operations offer supplementary origins of Zn pollution(Nanda and Agarwal 2016). One of the main pollutants discharged into the environment by mining, smelting, sewage sludge, industrial processes, and continuous use of Zn fertilisers is Zn. Zn can be found in soils in a variety of forms, primarily as free ions (Zn^2+^ and ZnOH^+^) or complexed with organic materials (Sagardoy et al. 2010). Higher Zn concentrations have been shown in studies to significantly lower seed germination, as demonstrated in *Vigna unguiculata*, *Cassia angustifolia*, and *Glycine max *(Nanda and Agarwal 2016; Gupta et al. 2016). Zn exposure causes notable alterations in the architecture of the root system (Disante et al. 2014). Primary root length has been reported to be shortened by Zn stress (Kranner and Kolville 2011). Plants of *Beta vulgaris* that have an overabundance of Zn had stunted root growth, appearing brown and with short lateral roots. The general metabolism was caused to shut down because there was a reduction in every phase of aerobic respiration in the roots due to a substantial excess of Zn (Sagardoy et al. 2010). Zn poisoning impacts the process of photosynthesis (Khan and Khan 2014). Photosystem II (PSII) efficiency parameters have been reported to decrease as Zn concentration rose (Anwar et al. 2015). For instance, toxic concentrations of Zn (43 ppm) in *Solanum lycopersicum* impeded plant growth and resulted in leaf chlorosis. This was because high concentrations of Zn have a negative impact on photosynthetic electron transport, cause the plasma membrane to break down, and reduce the permeability of the bio-membrane, all of which impair photosynthesis (Vijayaranjen and Mahalakxmi 2013). Zn stress also results in production of ROS although being a non-redox metal.Oxidative stress in *Oryza sativa* was triggered during Zn stress (Hosain et al. 2012).

## Heavy metal stress signaling in plants

### Calcium-dependent signaling pathway

Numerous investigations have revealed that the calcium ion (Ca^2+^) functions as a messenger in both the regular physiology of plants and their reactions to various environmental stressors (Himschoot et al. 2015). Ca^2+^ is essential for the response mechanism of plants and is used to transmit a variety of internal and external signals. Ca^2+^ channels can be found on the tonoplast, plasma membrane, and other organelles within plant cells (Tuteja 2009). For intricate interaction and signal transmission, stress stimuli regulate the cytosolic free Ca^2+^ concentration (Rudd and Franklin-Tong 2001). According to studies, the cytosolic Ca^2+^ concentration fluctuates during stressful situations due to either the release of intracellular Ca^2+^ storage or the import of Ca^2+^ from outside the cell (Steinhorst and Kudla 2014). The biological conversion of a signal mediated by a chemical messenger requires sensors that detect transient increases in Ca^2+^content (Steinhorst and Kudla 2014).

**Fig. 2:**
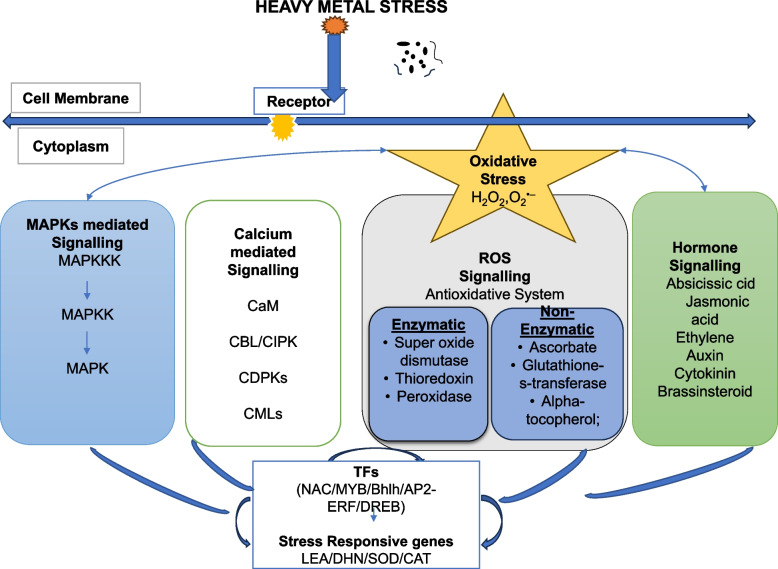
This figure illustrates key signaling pathways in plant responses to heavy metal stress: MAPK-mediated signaling activates stress-responsive genes for detoxification; calcium-mediated signaling regulates gene expression, ion transport, and enzyme activity using calcium as a messenger; ROS signaling enhances antioxidant defenses to combat oxidative damage; and hormone signaling modulates pathways (ABA, ethylene, SA, JA) to manage stress responses. These interconnected pathways form a complex network, enabling plants to sense heavy metal stress and activate coordinated defense mechanisms for survival and adaptation

All plant species share evolutionary conserved Ca^2+^sensing systems. Calmodulins (CaMs), calmodulin-like proteins, calcineurin B-like proteins (CBLs), and Ca^2+^dependent protein kinases (CDPKs) are just a few examples of the many different and extensive Ca^2+^ sensors found in plants that sense, interpret, and communicate changes in the cytosolic Ca^2+^ concentration for the response (Steinhorst and Kudla 2013). The “EF hand” helix-loop-helix motif is used by the majority of Ca^2+^ sensors to bind Ca^2+^ (Tuteja and Mahajan 2007). However, a small number of proteins bind Ca^2^^+^ without possessing EF-hand motifs. The cytoplasmic Ca^2+^ content varies in response to stimuli, and CDPKs act as sensors, relaying the signalling cascade downstream (Fig. 2) (Asano et al. 2012). A number of transcription factors are activated by the signal. Plant sensory proteins of the multigene family CDPKs directly bind calcium ions before phosphorylating substrates involved in stress signalling responses (Hamel et al. 2014). The specificity of the cellular response is determined by the kinetics and amplitude of Ca^2+^ fluctuations in response to signals.

When rice roots were subjected to both short-term and long-term Cr stress, transcript profiling revealed that CDPKs were involved because their activity rose as Cr concentration increased (Huang et al. 2014). Ca^2+^ was found to stimulate antioxidant enzymes and confer resistance against Cr stress in foxtail millet (Fang et al. 2014). According to studies, exogenous Ca^2+^ administration influences physiological and biochemical processes, hence mitigating the effects of heavy metal stress. Additionally, antioxidant enzymes such glutathione reductase, superoxide dismutase, and ascorbate peroxidase are activated more by exogenous Ca^2+^ (Ahmad et al. 2015).

### MAPK mediated signalling

The MAPK is a significant signalling module involved in numerous cellular and physiological processes in plants (Rao et al. 2010). The three-tier phospho-relay signalling mechanism is evolutionary conserved in the kingdom of plants, and MAPKs are serine/threonine kinases (Jonak et al. 2002). These signalling cascade phosphorylate numerous TFs such as ABRE, DREB, bZIP, MYB, MYC, NAC, and WRKY thus influencing metal stress response. As a result, they affect cellular processes related to stress response, growth, differentiation, and development. It has been suggested that MAPKs transmit stress signals, which in turn allow plants to activate their resistance mechanisms.

**Fig. 3:**
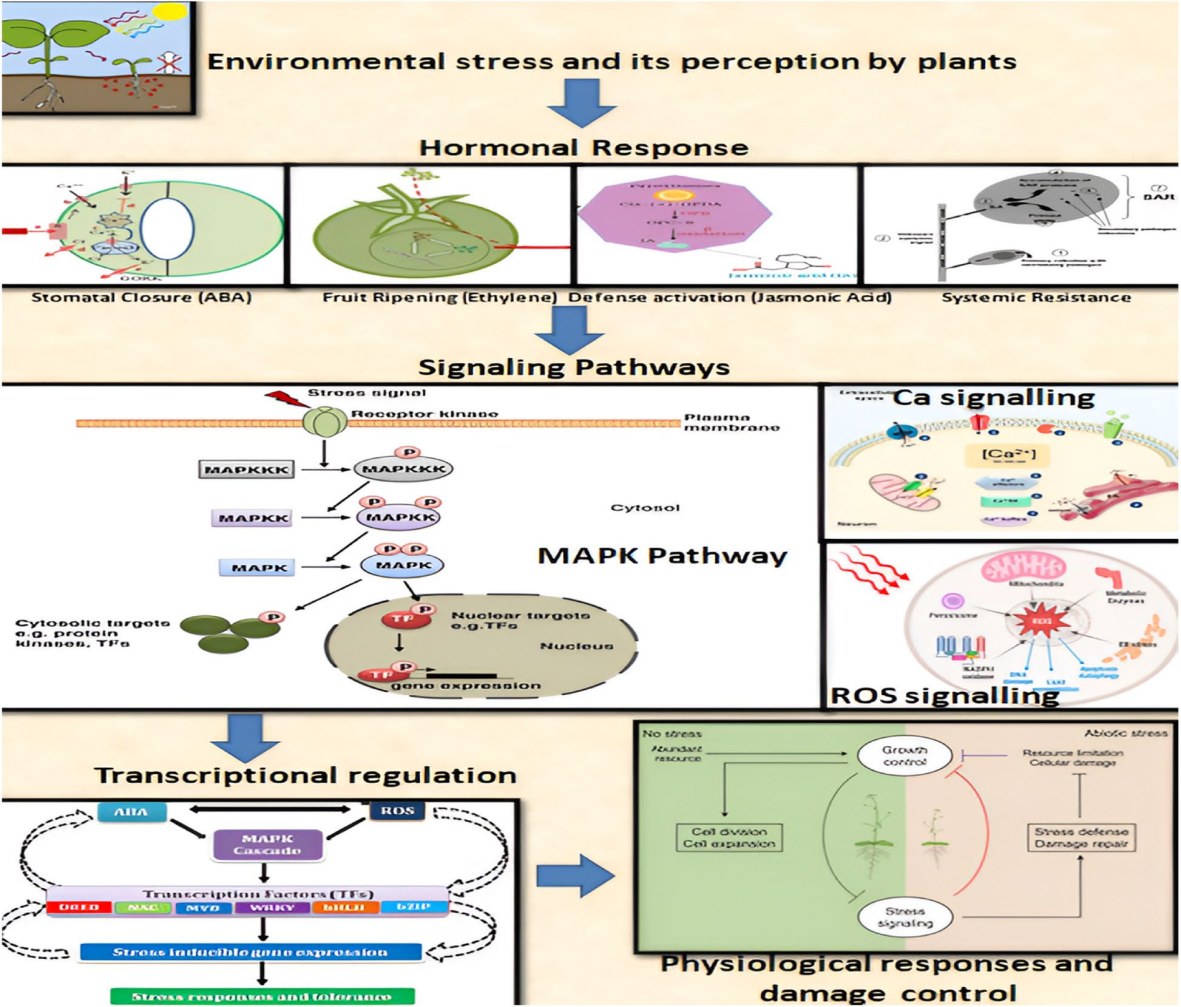
The figure illustrates the molecular mechanisms involved in plant stress responses. It emphasizes how specific receptors in plants detect biotic and abiotic stress, which causes NADPH oxidase to produce reactive oxygen species (ROS). Gene expression is modulated as a result of the ROS signals activating a cascade of mitogen-activated protein kinases (MAPKKK, MAPKK, and MAPK). This complex signaling network helps plants adapt to stress and maintain survival under harsh conditions

This signalling module triggers the plant's (Singh and Shah 2014) response to external stress cues and aids in initiating adaptive downstream signalling. MAP kinase cascades consist of MAPK, MAPKK, and MAPKKK components. This cascade has demonstrated unparalleled intricacy and has been conserved throughout the plant kingdom's history (Janitza et al. 2012). The MAPK module interprets various environmental stimuli, and when activated, MAPKs trigger a wide range of physiological and cellular reactions by activating cytosolic proteins and transcription factors (Fig. 3) (Samajova et al. 2013).The regulatory function of MAPKs in plant development, defence, and stress signalling is demonstrated by the transcriptional activation of these protein kinases in rice (Kim et al. 2003). Also in rice, Cd similarly activates the MBP kinase gene and MAPKs (OsMAPK2) (Yeh et al. 2004). In *Medicago sativa*, excess heavy metals (Cu and Cd) activate different MAPKs (Jonak et al. 2004). Similarly in Brassica juncea improved tolerance to As stress was observed as a result of the activation of MAPK cascades under As stress, which transduces metal stressmediated signals (Gupta et al. 2009).

### ROS signalling

Signal transduction is the plants’ ability to perceive stress stimuli and activate adaptive responses enabling them to survive in extremely harsh environments (Wrzaczek et al. 2013). One such indicator is the generation and build-up of reactive oxygen species (ROS) in various organelles, such as peroxisomes, mitochondria, and chloroplasts (mainly superoxide anion, hydrogen peroxide, hydroxyl radical, and singlet oxygen) (Wrzaczeket al. 2013). Reactive oxygen species (ROS) play a critical role in plant biology, serving as both signaling molecules that facilitate defense mechanisms and potential agents of toxicity. Understanding this dual function is essential for grasping their biological significance, especially in response to environmental stressors. In response to stressors like heavy metals, plants generate ROS, which act as signaling molecules. This process often begins with an “oxidative burst”, a rapid increase in ROS levels that signals the plant to activate defense-related genes, including transcription factors (Figs. 2 and 3) (Baxter et al. 2014; Hong et al. 2024). The signaling pathways triggered by ROS include the activation of mitogen-activated protein kinase (MAPK) pathways (Dvořák et al. 2021). These pathways are crucial for orchestrating responses to various stresses, enabling the plant to enhance its resilience against adverse conditions. The phenomenon known as the “ROS wave” illustrates how localized ROS production can initiate broader systemic responses throughout the plant. This coordinated signaling helps prepare other tissues for potential threats (Sahu et al. 2022), enhancing overall survival in challenging environments.

While low levels of ROS are beneficial for signaling, excessive accumulation can lead to oxidative stress, damaging vital cellular components such as proteins, lipids, and nucleic acids (Hong et al. 2024; Juan et al. 2021). This damage can trigger programmed cell death (PCD), which serves as a protective mechanism but can also compromise plant health if not properly controlled (Mansoor et al. 2022; Steffens 2014).Redox-sensitive phosphatases, histidine kinases, redox-sensitive transcription factors, and redox-regulated ion channels are examples of ROS receptors (Steffens 2014).Plants have evolved antioxidant systems to mitigate the harmful effects of excess ROS. A disruption in this balance—where the production of ROS exceeds the capacity for scavenging—can result in significant cellular damage and impaired physiological functions (Ali and Muday 2024; Huang et al. 2019). Numerous studies have demonstrated that exposure to heavy metals causes the production of reactive oxygen species (ROS), as evidenced by the build-up of malondialdehyde in plants that results in lipid peroxidation (Mansoor et al. 2023; Raiet al. 2015). Various studies have shown that differences in rice genotypes result in varying levels of reactive oxygen species production, potentially leading to distinct responses to arsenic exposure (Rai et al. 2015). Heavy metals like Cd disrupt chloroplast and mitochondrial metabolism, leading to lipid peroxidation and PCD in various plant species (Bi et al. 2009). Exposure to Hg leads to Ca^2^^+^ accumulation, reduces ROS generation, and activates MAPKs, supporting a plant's defence mechanism against Hg stress (Chen et al. 2014).

The dual role of ROS in plants highlights their complexity as both beneficial signaling molecules and potential sources of toxicity. Understanding how plants regulate ROS levels through various signaling pathways is crucial for comprehending their adaptive strategies and resilience mechanisms in response to environmental stresses. This balance is key to maintaining plant health and ensuring survival in harsh conditions.

### Hormone signalling

Hormones play a crucial role in regulating plant growth and development, with abscisic acid (ABA) being a significant participant in the plant's response to environmental stressors. Phytohormones are present in substantial quantities within plants and are essential for various physiological processes (Kazan 2015). ABA influences key functions such as bud dormancy and stomatal closure, while other hormones like auxin significantly impact growth, cytokinin delays senescence, gibberellin is involved in seed germination, and brassinosteroids regulate plant development and differentiation. Additionally, hormones such as ethylene (ET), jasmonic acid (JA), salicylic acid (SA), and ABA exhibit both antagonistic and synergistic effects that govern how plants respond to environmental stressors (Singh and Shah 2014). Notably, ET, JA, and SA are primarily associated with a plant's defense mechanisms against abiotic stresses (Lorenzo and Solano 2005). These phytohormones modulate the C-repeat binding factor pathway, with ET and JA activating critical signaling components and pathways, including JAZ proteins, EIN2, EIN3, and the AP2/ERF transcription factor gene family (Kazan 2015).

Research has demonstrated that exposure to JA enhances Cd stress tolerance in rice by increasing antioxidant responses and accumulation (Singh and Shah 2014). Similarly, in peas, Cd stress correlates with elevated ethylene levels (Vasilev et al. 2004). These findings indicate that plants exhibit differential responses to metal stress depending on the modulation of their phytohormone levels.

Abscisic acid (ABA) plays a key role in enhancing plant tolerance to heavy metal toxicityby regulating various physiological processesRecent research has highlighted several mechanisms by which ABA alleviates the stress caused by heavy metals such as cadmium (Cd), lead (Pb), and arsenic (As). A study showed that ABA application modulates the expression of metal transport genes like IRT1 and HMA, reducing heavy metal uptake and translocation in plants (Zhao et al. 2023). In ramie (*Boehmeria nivea*), Cd exposure combined with ABA treatment significantly induced BnPCS1 expression, a gene involved in phytochelatin synthesis and metal detoxification (Hu et al. 2020). This suggests that ABA not only enhances antioxidant responses but also promotes the chelation of metal ions, thereby mitigating their toxic effects on plant cells.

Moreover, a comparative transcriptomic analysis between *Arabidopsis thaliana* (Pb-sensitive) and Hirschfeldi aincana (Pb-tolerant) revealed that genes related to ABA biosynthesis were upregulated in response to Pb exposure in the tolerant species, indicating a potential adaptive mechanism involving ABA signaling pathways (Hu et al. 2020). Additionally, exogenous ABA has been shown to inhibit Cd uptake, translocation and accumulation, and promoting Cd chelation and efflux in *Arabidopsis thaliana*, further supporting its protective role against heavy metal toxicity (Meng et al. 2022). Recent studies highlight the crucial role of abscisic acid in enhancing plant resilience to heavy metal stress. By regulating gene expression related to metal uptake and promoting antioxidant defenses, ABA serves as a vital component in developing strategies for improving crop tolerance to heavy metal contamination. Continued research into the molecular mechanisms underlying ABA's action will be essential for advancing agricultural practices aimed at mitigating heavy metal toxicity.

## Multiomics and bioinformatic tools to decipher about heavy metal defence mechanism

### Genomics

Genomics is the study of genes and their interactions in specific physiological states. It involves sequencing genes and their surrounding regions, as well as describing their structure and function (Jamla et al. 2021). Genomics has transformed plant research by providing valuable insights into plant biology, breeding, and agriculture.

One key application of genomics in plant research is understanding plant biology at the molecular level. By sequencing plant genomes, we can identify genes responsible for important traits such as yield, disease resistance, and stress tolerance. This information is essential for developing improved crop varieties through genetic engineering and breeding programs (Bashir et al. 2021; Huet al. 2018; Prohens 2011; Mansoor et al. 2024a). Genomics has also illuminated the evolutionary history of plants, allowing us to trace the origins of different crop species and understand how they have adapted to various environments over time. Comparative genomics helps us identify genetic similarities and differences between plant species, which is valuable for crop improvement efforts (Brozynska et al. 2016; Morrell et al. 2012; Khan et al. 2024).

Genomics has been crucial in uncovering the genetic basis of plant diseases and pests (Khan et al. 2023). By sequencing the genomes of pathogens and pests, we can create new strategies for disease control and pest management, such as breeding for resistance or developing targeted treatments (Keon et al. 2003; Lucas 2011; Stukenbrock 2013). Another significant application of genomics in plant research involves studying gene expression and regulation. Transcriptomic and epigenomic studies have revealed complex regulatory networks that control gene activity in plants, providing insights into how plants respond to environmental changes (Imadi et al. 2015; Liu et al. 2015; Urano et al. 2010). In rice, 46 heavy metal-associated proteins (HMPs) were identified; in Arabidopsis, 55 were found; and in Solanum tuberosum, 36 genes across 6 subfamilies containing the HMA domain were identified, revealing diverse gene structures and motifs (Li et al. 2020; He et al. 2020).

In plants, the genomic response to heavy metal stress is mainly influenced by chromatin structure, late-responding genes like transcription factors, and specific DNA sequences that regulate gene expression (Bohnert, et al. 2006; Khalid et al. 2019; Jamla et al. 2021). Common bioinformatic tools used in plant genomics research include a variety of applications. For example, BLAST is a widely used tool that helps compare nucleotide or protein sequences against databases to find homologous counterparts (Coordinators). MAKER (Campbell et al. 2014)and AUGUSTUS (Brůna et al. 2021)are essential for genome annotation through gene prediction and functional annotation. Clustal Omega and MUSCLE support multiple sequence alignments, helping identify conserved regions and evolutionary relationships among different plant species (Higgins and Sharp 1988). Genome browsers like Integrative Genomics Viewer (IGV) and UCSC Genome Browser provide graphical insights into genomic features and gene expression patterns (Freese et al. 2016; Rangwala et al. 2024). Additionally, phylogenetic tools such as MEGA and BEAST are significant for understanding evolutionary lineages and genetic diversity among plant populations through genomic sequence analyses (De Bruyn et al. 2014).

### Transcriptomics

The genomics approach helps identify target genes within an organism, but conducting expression analysis is essential to understand the functional roles of these genes (Zhou and Zheng. 2022). Transcriptomic profiling improves our understanding of how organisms respond in specific physiological states or under stress. Regulatory gene expression at the molecular level plays a critical role in biological processes, affecting plant development and their ability to withstand heavy metal stress (Raza et al. 2022). Stressors activate many genes and proteins to create signaling pathways that enhance stress tolerance. These genes can be divided into two categories: regulatory and functional. Regulatory genes encode transcription factors (TFs) that control stress-responsive genes, forming a gene network (Feng et al. 2023; Mansoor et al. 2024a, b). Functional genes encode metabolic compounds like alcohols, amines, and sugars, which are vital for heavy metal stress (HMS) tolerance (Singh et al. 2016a, b). Research shows that a single TF can regulate multiple target genes by binding to specific cis-acting elements in their promoters (Zhou and Zheng 2022).

Transcriptome analysis has revealed the functions of genes related to metal tolerance in several plant species. For example, a regulatory gene similar to FER, which is responsible for iron uptake in tomatoes, was identified (Liang et al. 2013). This FER-like Deficiency Induced Transcription Factor (FIT) also plays a significant role in Arabidopsis under iron deficiency (Yuan et al. 2005). Heavy metal stress in A. thaliana showed downregulation of dehydration-responsive transcription factors in roots, correlating with osmotic stress-regulating genes. These identified TFs likely helped normalize the osmotic potential of plants and reduce the flow of contaminated water through them (Nakashima and Yamaguchi-Shinozaki. 2006). RNA sequencing in the roots of wheat plants under Cd (Cd) stress revealed transcriptional changes that provided insights into gene reprogramming (Xiao et al. 2019). A meta-transcriptomic study reported 838 differentially expressed genes (DEGs) across multiple datasets under Cd stress in Gramineae species (Fan et al. 2021). Comparative expression studies on Indian mustard identified arsenic-responsive genes involved in vital life processes and stress tolerance mechanisms (Thakur et al. 2019). A comparative transcriptome analysis found 3,696 DEGs in Tall fescue under lead (Pb) stress (Li et al. 2017a, b), while a similar study in tea plants revealed candidate genes induced by aluminum stress (Li et al. 2017a, b). A comparative transcriptomic analysis of Salix babylonica under cobalt stress identified 1,165 DEGs in roots and approximately 107 TFs from these DEGs belonging to ERC and NAC families (Wang et al. 2020a, b, c).

**Fig. 4:**
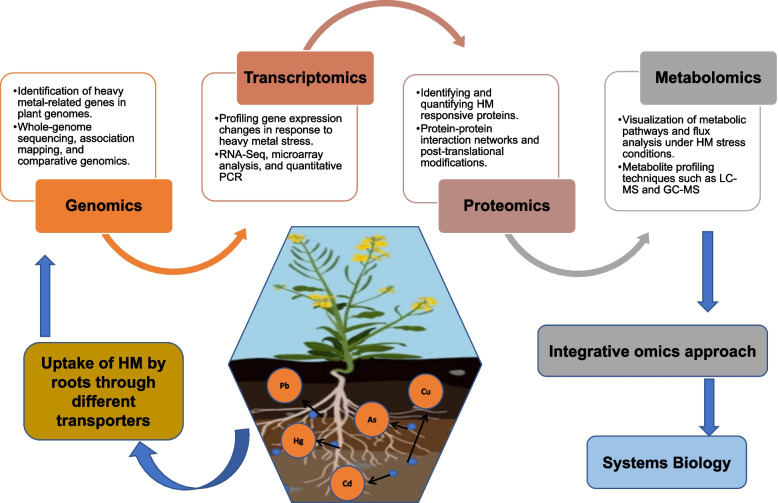
This figure highlights the integration of multiomics approaches for understanding heavy metal stress tolerance in plants. Genomics identifies tolerance-related genes through comparative genomics and genome-wide association studies. Transcriptomics analyzes gene expression changes under stress using RNA sequencing. Proteomics identifies and quantifies proteins involved in heavy metal responses via techniques like mass spectrometry. Metabolomics examines metabolite changes and pathways using GC–MS and LC–MS. Bioinformatics and systems biology integrate these datasets to provide a comprehensive view of biological responses, uncovering molecular mechanisms, biomarkers, and targets to enhance stress resistance. This holistic approach advances strategies for mitigating heavy metal toxicity in plants

Understanding the complex mechanisms that govern gene expression is crucial in plant biology, especially for enhancing crop resilience against harsh conditions like heavy metal stress (Nakashima et al. 2009). To effectively tackle this challenge, single-cell transcriptome analysis is essential as it provides insights into the toxicity caused by metals and metalloids. Such insights are foundational for developing new strategies to regulate ion flux, signal transduction, and metabolic pathways (Ghuge et al. 2023). Combining transcriptomics data from various studies enhances our understanding of functional genomics and supports efforts in molecular breeding, phytoremediation, genetic engineering, and metal chelation pathways (Phurailatpam et al. 2022). Furthermore, integrating transcriptomics with other omics datasets using advanced tools is vital for uncovering hidden connections and extracting actionable insights that lead to innovative approaches (Fig. 4) (Jamla et al. 2021; Mansoor et al. 2024b).

The transcriptomic approach allows exploration of gene expression patterns across different developmental stages, environmental conditions, and stress responses in plants. This reveals the complexities of gene regulatory networks. Identifying differentially expressed genes clarifies their specific roles in plant growth, development, and responses to stressors (Ma et al. 2017; Wang et al. 2020a, b, c; Mansoor et al. 2024b). By examining alternative splicing events and the resulting variety of mRNA isoforms from a single gene, transcriptomics helps uncover the functional diversity of genes and their protein products (Nejat, Ramalingam and Mantri 2018; Wei et al. 2021). Comparative transcriptomic analyses deepen our understanding by revealing evolutionary relationships, genetic variations, and differences in gene expression among various plant species or genotypes. This ultimately enhances research into plant adaptation, diversification, and evolution (Do Amaral et al. 2016; Ma et al. 2017; Wang et al. 2020a, b, c; Wei et al. 2021).

DESeq2 and edgeR are key tools for conducting differential gene expression analysis, identifying significantly altered genes under different experimental conditions in plants (Niedziela et al. 2022). Cufflinks is commonly used for transcript assembly and quantification, especially for de novo transcriptome assembly from RNA-seq data. It aids in predicting transcript structures and estimating abundance in plants (Ghosh and Chan 2016). Alignment tools like MapSplice and STAR are essential for accurately mapping RNA-seq reads to the plant reference genome, facilitating precise identification of key genomic features (Dobin et al. 2013). Tools such as DAVID and g:Profiler help functionally annotate differentially expressed plant genes by categorizing them into biological processes, molecular functions, and cellular components, shedding light on gene functionality in plant transcriptomes (Dennis et al. 2003; Reimand et al. 2016). WGCNA is used to construct gene co-expression networks to uncover co-expressed gene modules and explore gene–gene interactions within plant regulatory networks (Zhang and Wong 2022). Additionally, pathway analysis tools like KEGG help reveal enriched biological pathways containing differentially expressed genes, providing insights into the functional implications of gene expression changes within specific plant pathways (Altman et al. 2013; Masoudi-Nejad et al. 2007).

### Proteomics

Proteomics provides a detailed view of the proteins present in living organisms at any given time, making it an essential tool for studying complex cellular pathways at the molecular level (Mehmood et al. 2021). Recently, it has become a key method for gathering data on how plants survive and adapt to the toxic effects of metals and metalloids (Dabral et al. 2024). Proteins play a crucial role in plant responses to stress, including adaptation to heavy metal exposure, by helping restore functional protein structures (Phurailatpam et al. 2022). This process is vital for maintaining redox homeostasis, which is essential for plant survival under heavy metal stress conditions (Wang et al. 2012).

Proteomics technology has rapidly advanced from first-generation techniques like two-dimensional electrophoresis-mass spectrometry to second-generation methods involving isobaric/isotopic tagging. It has progressed to third-generation approaches such as shotgun and gel/label-free techniques, and finally to fourth-generation methods like mass western, targeted, and SRM/MRM approaches (Jorrin-Novo et al. 2019).Several studies have examined how plants, including rapeseed and maize, respond to Cd (Cd) stress. In rapeseed, about 672 proteins were detected in the xylem sap using LC–MS analysis. These proteins help enhance Cd tolerance by acting as chelating agents (Luo and Zhang 2019). In maize seedlings, a study using the iTRAQ method identified differentially abundant proteins (DAPs) in the roots. These proteins were linked to various functions such as cellular metabolism and scavenging reactive oxygen species (ROS). Notably, the GST protein was emphasized for its role in providing Cd tolerance (Wen et al. 2019).

The effect of selenium (Se) on heavy metal toxicity, especially arsenic (As) toxicity, has been studied in rice and pepper plants. In rice, a comprehensive study combined transcriptomics and proteomics to understand Se-mediated tolerance to As toxicity. Treatment with both Se and As led to the upregulation of specific Glutathione S-transferases (GSTs) and other proteins, which helped reduce oxidative stress (Chauhan et al. 2020). Similarly, in pepper seedlings, LC–MS-based proteomic analysis explored how Se mitigates heavy metal toxicity. This analysis revealed 172 upregulated proteins, including heat shock proteins among the DAPs, which helped the plants combat heavy metal toxicity (Zhang et al. 2019a, b).The impact of manganese (Mn) toxicity on tomato plant roots was studied using two proteomic approaches: 2D electrophoresis (2DE) and the shotgun method. The 2DE method identified 54 DAPs, while the shotgun method found 118 DAPs, with 7 proteins common to both methods. These proteins were involved in various metabolic processes that affected root cell wall integrity and energy production pathways (Ceballos Laita et al. 2018).

Plants detect and respond to heavy metal toxicity by initiating complex intracellular signaling cascades that activate defense mechanisms (Singh et al. 2016a, b). Understanding how protein levels change within cells under stress conditions is crucial. Detailed research into individual cell or organelle proteomics offers valuable insights into how plants respond to heavy metal stress (Phurailatpam et al. 2022). This knowledge is essential for developing plants with greater tolerance to heavy metals, thereby contributing to sustainable agriculture and environmental remediation efforts (Dabral et al. 2024).MaxQuant is a widely used tool in proteomics research for analyzing mass spectrometry data. It helps quantify protein expression levels, detect post-translational modifications, and examine protein–protein interactions in plants. Perseus is software that works alongside MaxQuant for statistical analyses, data visualization, and interpreting proteomics data in plant studies (Rudolph 2019; Tyanovaet al. 2016). Scaffold is a bioinformatics tool designed for protein identification and validation in mass spectrometry-based proteomics studies. It helps organize and analyze proteomics data, including protein quantification and peptide identification in plant samples (Boetzeret al. 2011). Additionally, STRING-DB is a bioinformatics database that predicts protein–protein interactions and functional relationships. It enables the construction of protein interaction networks, annotation of protein functions, and exploration of biological pathways in plant proteomics research (Franceschini et al. 2012; Shakirova et al. 2022).

### Metabolomics

Metabolomics is a growing field focused on detecting and quantifying a wide range of low molecular weight molecules (less than 1 kDa), including metabolites (Raza 2020). This discipline is notable for its broad scope and advanced techniques, gaining popularity and creating extensive databases (Feng et al. 2022). It provides valuable insights into how plants respond internally to various environmental conditions. Metabolomics helps capture and analyze the changes in plant responses under both stressed and normal conditions, offering important data for measuring and understanding the plant's genetic and environmental reactions (Feng et al. 2022). Some methods used to study plant metabolic responses include inductively coupled plasma mass spectrometry (ICP-MS) and liquid and gas chromatography coupled with mass spectrometry (LC–MS, GC–MS), as well as nuclear magnetic resonance spectroscopy (NMR) (Mashabela et al. 2023).

Metabolomic analysis has been used to assess the impact of Cd (Cd) stress on different plant species, including rice and amaranth. In amaranth, researchers used LC–MS and HPLC techniques to identify around 41 significantly different metabolites (SDMs), with 12 linked to key metabolic pathways under Cd stress (Xie et al. 2019). Similarly, in rice, comparable techniques were employed to investigate the effects of both Cd and copper (Cu) toxicity (Navarro-Reig et al. 2017). Out of 112 identified metabolites, 97 were directly associated with Cd toxicity, particularly affecting cellular metabolism like amino acids and secondary pathways. Cu stress was studied in cucumber plants using LC–MS and GC-TOF–MS approaches, revealing 228 primary and secondary metabolites that impacted carbohydrate and ascorbate metabolism pathways (Zhao et al. 2018). In a study on selenium tolerance in celery seedlings, researchers identified 1,774 metabolites and 237 SDMs using UHPLC/LC–MS, highlighting metabolic pathways essential for Se tolerance (Zhang et al. 2019a, b).Zn stress was examined in tea plants regarding both excess and deficiency, showing significant impacts on flavonoid and carbohydrate metabolism pathways (Zhang et al. 2017a, b). Arsenic stress was studied in two genotypes of green chiretta. The wild type accumulated less arsenic than cultivated lines. Using HPLC, researchers quantified metabolites, revealing distinct variations in secondary metabolites, oxidative enzymes, and nutrient uptake during detoxification under arsenic stress (Zhang et al. 2019a, b). Using metabolomics allows for the construction of metabolite networks under stress conditions (both biotic and abiotic), which can enhance crop improvement efforts. This approach is useful in areas such as metabolomics-assisted breeding, developing transgenic plants, drug discovery, extraction processes, and mutant characterization (Razzaq et al. 2019). Recent technological advancements have significantly boosted research on metabolite profiling. Innovations in instrument design and the use of mass spectrometry and chromatography principles have greatly improved the characterization and quantification of these metabolites (Mashabela et al. 2023).

Bioinformatics tools are indispensable in modern plant science, particularly for unraveling the molecular mechanisms underlying plant stress responses. These tools enable researchers to process, analyze, and interpret complex datasets across multiple -omics platforms, providing insights into how plants adapt to abiotic and biotic stresses. Below is an improved discussion emphasizing their practical applications in analyzing plant stress responses.One of the key bioinformatics tools for studying plant stress responses is MetaboAnalyst. It is a versatile platform for metabolomics data analysis. Its applications in plant stress research include pathway mapping which helps in identifying stress-related metabolic pathways activated during abiotic stresses like drought or salinity. Comparative Analysis can be performed to visualize metabolite changes across stress conditions and identify biomarkers of tolerance or susceptibility. Metabolomics can be integrated with transcriptomics or proteomics to uncover multi-level regulatory mechanisms of stress responses (Xia and Wishart 2011). Another tool XCMS specializes in untargeted metabolomics analysis using high-resolution mass spectrometry. It contributes to stress biology in the detection of stress-Induced novel metabolites produced during oxidative stress or heavy metal exposure. It also helps in temporal monitoring of dynamic changes in metabolite profiles over the course of a stress response (Vinay et al. 2021).

MZmine is an open-source software which supports comprehensive metabolomic workflows. In plant stress studies, it is used for quantification of stress biomarkers like proline or antioxidants under drought or heat stress and the chromatographic alignment across samples for ensuring accurate comparisons between stressed and control samples (Pluskal et al. 2020). MetFrag facilitates the identification of unknown metabolites by matching experimental MS/MS data with theoretical fragmentation patterns. Its relevance includes characterizing novel stress-responsive compounds which uncover secondary metabolites involved in plant defense mechanisms. It is also involved in structural elucidation providing insights into the chemical nature of compounds that mediate tolerance to abiotic stresses such as salinity or cold (Ruttkies et al. 2019). MassBank, a repository of mass spectral data, aids in validation of metabolite identification by comparing experimental spectra with reference data to confirm the identity of metabolites linked to stress responses. It also facilitates cross-species comparisons, investigating conserved metabolic responses across different plant species under similar stress conditions (Ara et al. 2021). Plant PhysioSpace offers a robust framework for quantitative analysis of plant stress responses by comparing experimental data with precomputed models. Its applications include cross-species stress analysis translating stress response data between species to identify conserved adaptive mechanisms. It also helps in quantitative trait assessment by evaluating the effects of genetic modifications or treatments on stress tolerance, such as in heat-stressed wheat or biotic-stressed crops (Lenz et al. 2013; Hadizadeh Esfahani et al. 2021).

CRISPR/Cas9 integration with bioinformatics tools has now enabled precise editing of genes involved in abiotic stress tolerance. It is employed in target identification by integrating transcriptomic and metabolomic data to identify candidate genes for editing. It has paved way for the development of stress-tolerant crops, for example, editing genes like OsBADH2 or SAPK2 to enhance tolerance against drought, salinity, and heat (Kumar etal. 2023, Ambrosino et al. 2020). Advanced bioinformatic platforms such as STRING and KEGG facilitate pathway mapping and integration across omics layers (genomics, transcriptomics, proteomics, and metabolomics). These approaches are critical for modeling stress pathways by constructing predictive models of oxidative stress-related pathways. It also integrates data across platforms by combining datasets from RNA sequencing, mass spectrometry, and protein profiling to gain holistic insights (Ambrosino et al. 2020; Zhang et al. 2024).

The application of bioinformatics tools in plant stress research continues to evolve with advancements in artificial intelligence (AI) and machine learning (ML). AI-assisted tools can predict gene-environment interactions and identify novel targets for genetic engineering (Zhang et al. 2024). ML algorithms can analyze large-scale multi-omics datasets to uncover hidden patterns and correlations related to stress adaptation (Upadhyay et al. 2017; Laha et al. 2020).Bioinformatics tools are revolutionizing our understanding of plant stress responses by enabling high-throughput analysis, integrative modeling, and actionable insights into molecular mechanisms. By leveraging these tools, researchers can develop innovative strategies for engineering crops with enhanced resilience to environmental stresses.

## Approaches of plants to mitigate heavy metal toxicity

Heavy metal toxicity in plants presents considerable challenges to agricultural productivity, necessitating the exploration of effective mitigation strategies (El-Sappah et al. 2024). Agronomic approaches, particularly phytoremediation, leverage hyperaccumulator species such as *Sedum alfredii* to extract and sequester heavy metals from contaminated soils (Wan et al. 2024). Additionally, practices like crop rotation and the application of organic amendments, including biochar and farm manure, contribute to enhanced soil health and reduced bioavailability of heavy metals (Hassan Bashir et al. 2024). Traditional methods, such as foliar applications of nutrients like zinc sulfate (ZnSO_4_), have demonstrated efficacy in physiologically blocking heavy metal uptake, thereby alleviating toxicity in staple crops such as rice and wheat (Wan et al. 2024). Recent innovations include the utilization of nanoparticles to improve plant tolerance by modulating metal distribution and detoxifying reactive oxygen species (ROS), which ultimately enhances growth and yield (El-Sappah et al. 2024). Furthermore, integrating molecular breeding techniques with conventional methods aims to develop cultivars that exhibit low heavy metal accumulation while maintaining high productivity levels. These combined approaches underscore the importance of fusing traditional agricultural practices with modern scientific advancements to promote sustainable farming in environments affected by heavy metal contamination. The integration of these methods not only addresses the immediate impacts of heavy metal toxicity but also contributes to long-term soil restoration and food security (Ejaz et al. 2023).

### Phytoremediation

Phytoremediation is a highly effective and cost-efficient method for removing heavy metals from contaminated soils, utilizing the natural capabilities of plants and their associated microorganisms to mitigate environmental pollutants (Sarwar et al. 2017; Rai et al. 2021). In this process, plants absorb heavy metals through their roots, where the metals may either remain in root tissues (phytoimmobilization) or be transported to aerial parts via xylem vessels using symplastic and/or apoplastic pathways. Within the shoots, metals are typically stored in vacuoles, cellular organelles with low metabolic activity, which protect essential cellular functions from metal toxicity. This mechanism is particularly vital in hyperaccumulator plants (Rai et al. 2021). The phytoremediation process involves five key steps: mobilizing metals in the rhizosphere, absorbing metal ions through roots, translocating metals to aboveground parts, sequestering metals within plant tissues, and ensuring plant tolerance to heavy metal stress (Sarwar et al. 2017). The success of this process depends significantly on a plant's tolerance to metal stress, as higher tolerance enhances metal accumulation with minimal adverse effects on plant health. This tolerance is influenced by mechanisms such as binding metals to cell walls, transporting metal ions into vacuoles, and forming complexes through chelation with proteins and peptides (Setia et al. 2008).

Microorganisms associated with plant roots play a crucial role in enhancing phytoremediation efficiency. These microorganisms secrete organic acids—such as citric, tartaric, and gluconic acids—that increase the bioavailability of heavy metals by binding to them in the soil solution and facilitating their absorption by plant roots (Shah and Daverey 2020). Certain bacteria, including Bacillus subtilis, Lactobacillus species, and Pseudomonas aeruginosa, produce secondary metabolites that aid in removing hazardous heavy metal ions from the soil matrix. These metabolites include biosurfactants such as lipopeptides, glycolipids, and fatty acids, which enhance solubilization and mobilization of metal ions for plant uptake (Shah and Daverey 2020; Araújo et al. 2019).

Various plant species are employed in phytoremediation strategies to address heavy metal pollution. For example, Brassica species are widely used due to their effectiveness in techniques such as phytovolatilization, phytoextraction, and phytostabilization (Lv et al. 2013; Thakur et al. 2016). Brassica napus has demonstrated significant bioconcentration of Zn, Pb, Cu, and Cd from polluted soils (Ali et al. 2013), while Thlaspi caerulescens and bladder campion have shown potential for remediating soils contaminated with Zn and Cd (Brown et al. 1994). Additionally, Jatropha curcas has been identified as a promising crop for coal fly ash remediation (Jamil et al. 2009). Field studies with *Solanum nigrum* L. on Cd-contaminated soils revealed significant biomass output; double cropping enhanced Cd extraction while fertilization had limited impact (Ji et al. 2011). The use of Na_2_-EDTA solutions has also been shown to improve the solubility of Cd, Zn, and Pb in soil solutions, promoting their absorption in rainbow pink shoots. Rainbow pink exhibited potential for Cd and Pb phytoextraction, while vetiver grass demonstrated resilience without growth inhibition despite contamination levels, making it suitable for phytostabilization in multi-metal polluted soils (Lai and Chen 2004).

In regions like the Belgian and Dutch Campine areas—where soils are moderately contaminated with Pb, Zn, and copper—traditional agriculture faces challenges. To address this issue, energy maize is being cultivated for “phytoattenuation”, a strategy aimed at gradually reducing pollution levels while providing an alternative income source for farmers. This approach not only helps mitigate soil contamination but also generates renewable energy. Energy maize farming can produce approximately 33,000–46,000 kWh of renewable energy per hectare annually while significantly reducing CO_2_ emissions from coal-fired power plants by up to 21 × 10^3^ kg ha^-1^ y ^−^^1^. Although Cd and Pb removal rates are negligible in this method, Zn removal is more pronounced with an annual reduction of 0.4–0.7 mg kg^-1^ in the topsoil layer (Meers et al. 2010).

Recent advancements in phytoremediation highlight innovative approaches to combat heavy metal toxicity. For instance, *Helianthus annuus* (sunflower) combined with heavy metal-resistant bacteria like *Ralstoniaeutropha* and Chrysiobacteriumhumi significantly reduced Zn and Cd bioaccumulation by up to 67%, while improving rhizosphere bacterial diversity and plant stabilization under heavy metal stress (Park and Oh 2023). Similarly, *Robinia pseudoacacia* was used to remediate sterile dumps contaminated with heavy metals, leveraging its deep root system and high biomass production for long-term remediation in poor soil conditions (Anne et al. 2023). The bacterium *Raoultella* sp. X13 enhanced Cd phytoremediation by solubilizing phosphates and producing siderophores, which improved plant growth and reduced Cd bioavailability in the soil (Yan et al. 2020). Advances in CRISPR technology boosted the efficiency of Brassica juncea and Lupinus albus, increasing As phytoaccumulation by 85% and Hg accumulation by 45% through enhanced expression of metal-binding proteins like phytochelatins and metallothioneins (Khatoon et al. 2024). Constructed wetlands featuring aquatic plants such as water hyacinth (Eichhornia crassipes) effectively absorbed Cr and Pbfrom wastewater, restoring aquatic ecosystems (Kabir et al. 2022). Energy maize cultivation in Europe achieved dual benefits by reducing Zn levels in contaminated soils by 0.4–0.7 mg/kg annually while generating renewable energy, reducing CO_2_ emissions by up to 21 × 10^3^ kg/ha/year (Yuliasni et al. 2023). Additionally, the use of ethylenediaminetetraacetic acid (EDTA) enhanced Pb uptake in plants grown on Pb-contaminated soils by increasing metal solubility and promoting higher accumulation in plant tissues (Gul et al. 2020). These projects demonstrate the potential of integrating microbial assistance, genetic engineering, and innovative field applications to enhance phytoremediation efficiency while promoting environmental sustainability.

### Physiological approaches

According to Ovecka and Takac (2014), the growth and development of plants are critically dependent on the continuous absorption of various solutes from the rhizosphere, followed by their distribution throughout the plant. In addition to essential nutrients and water, plants can absorb heavy metal ions present in the soil, which may accumulate into their tissues. The response of plants to heavy metals varies significantly between susceptible and resistant species (Pavlovkin et al. 2009). Heavy metal ions can directly affect substance transport and signaling pathways through interactions with the plasma membrane, which serves as the primary physiological barrier (Pavlovkin et al. 2009). To mitigate metal ion absorption, resistant plants employ various strategies, including the buffering capacity of their roots to precipitate and complex these ions near the root zone (Reichman et al. 2002). The anatomical characteristics of roots are influenced by cell wall properties, which act as mechanical barriers to prevent heavy metal accumulation within cells. The root apoplast functions as a dynamic barrier that inhibits heavy metal uptake and reduces oxidative stress (Wang et al. 2004).

Plants require certain metals, such as Fe, Cu, Mn, Zn, and Co, at specific concentrations; however, excessive amounts can be toxic (Arif et al. 2016). Conversely, metals such as Ag, Al, As, Cd, Cr, Cs, Hg, Pb, Sr, and U can induce phytotoxicity even at low concentrations (Nagajyoti et al. 2010). To cope with metal stress, plants utilize a variety of mechanisms, including complexation, exclusion, compartmentalization, and the synthesis of chelators and proteins that bind to metals. A common intracellular detoxification mechanism is chelation, primarily mediated by thiol-containing molecules such as glutathione (GSH), phytochelatins (PCs), and metallothioneins (MTs). Elevated levels of heavy metals lead to oxidative stress through the generation of reactive oxygen species (ROS) (Sharma et al. 2012). GSH plays a critical role in synthesizing phytochelatins that facilitate metal detoxification. Furthermore, the regulation of ROS levels through the ascorbate–glutathione (AsA–GSH) pathway enhances plant resistance to metal-induced oxidative stress (Hasan et al. 2017). These systems are essential for enabling plants to withstand conditions of metal and metalloid stress effectively. The interplay between heavy metals and plant physiology is complex. Plants have evolved sophisticated mechanisms for metal uptake and detoxification that include physical barriers at the root level and biochemical strategies for managing oxidative stress and maintaining homeostasis. Understanding these mechanisms is crucial for developing effective strategies for phytoremediation and enhancing plant resilience in contaminated environments.

### Biotechnological approaches

**Fig. 5:**
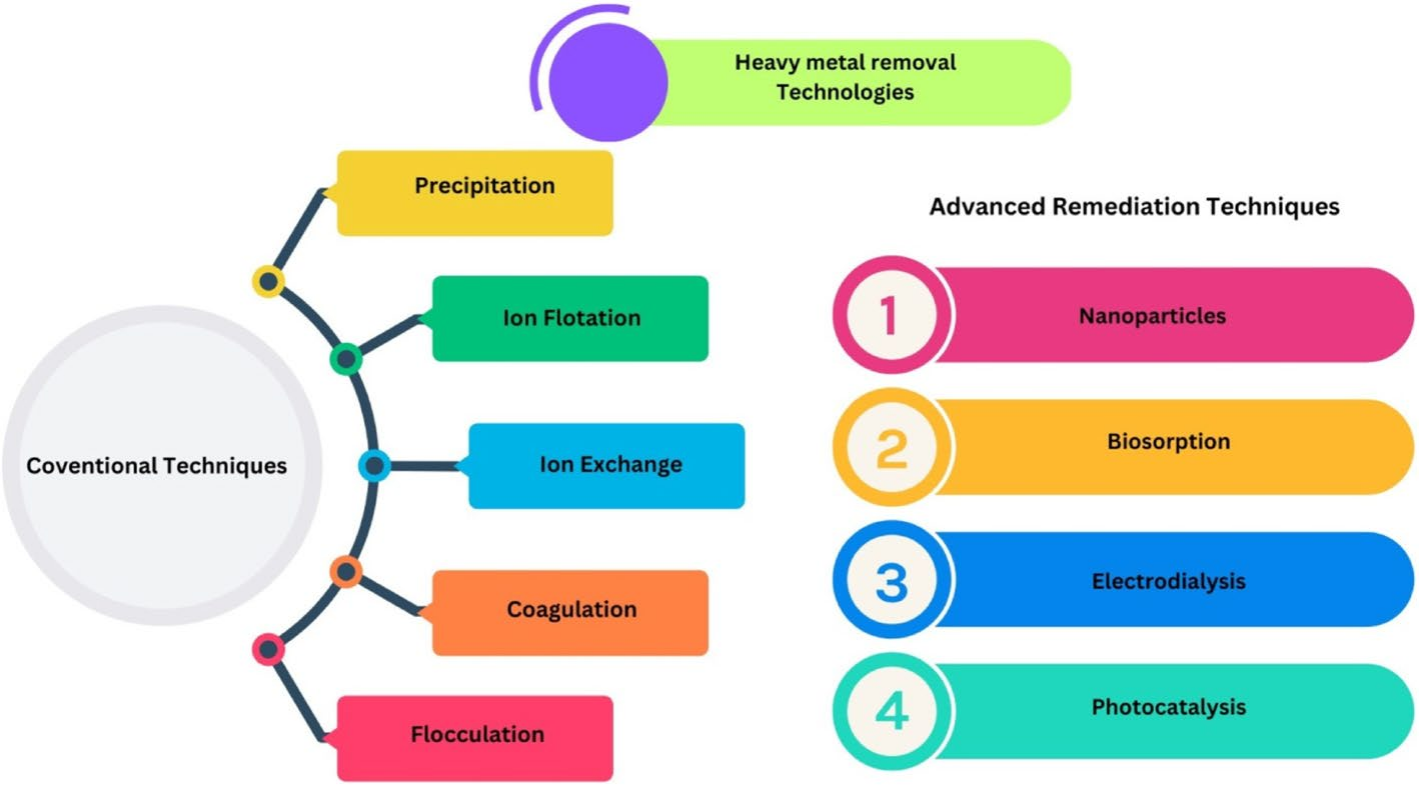
Figure shows different conventional and advanced remediation techniques

According to Ovecka and Takac (2014), the advent of multi-omics technology has yielded significant insights into the molecular pathways that underlie plant responses to heavy metal toxicity. The intricate character of plant defense systems has been highlighted by several studies that have shown considerable changes in metabolite profiles, protein levels, and gene expression in response to heavy metal stress (Bona et al. 2007; Alvarez et al. 2009). Nowadays, the focus is on developing plants that can tolerate heavy metal stress without sacrificing yield by identifying important genes and pathways involved in stress response and avoidance (Ovecka and Takac 2014). This information might lead to the creation of robust crop types that are more adapted to thriving in polluted areas (Bona et al. 2007). Genetic engineering is supporting the developing field of phytoremediation, which aims to solve metal contamination by modifying plant traits such as metal absorption, transport, and tolerance. Metallothionein for Cd tolerance and mercuric ion reduction for resistance and phytoextraction are two rare examples of effective use. Promising directions for enhancing phytoremediation can be found in the further investigation of putative transformation-related genes (Kärenlampi et al. 2000). The application of CRISPR/Cas9 technology to improve the phytoremediation of heavy metals (HMs) by aromatic and decorative plants appears promising. These plants provide a viable and affordable option for HM remediation because to their quick development and significant biomass output. Without raising concerns about genetic modification, CRISPR/Cas9 editing can enhance phytoremediation ability by targeting specific genes (Nayeri et al. 2023). This approach offers innovative strategies for long-term HM management using genetically engineered plants by thoroughly examining regulatory concerns, molecular aspects of plant tolerance to heavy metals, and protective mechanisms (Nayeri et al. 2023). Transgenic tobacco plants expressing *Arabidopsis thaliana*'s AtACR2 show enhanced arsenic tolerance in comparison to wild-type plants, since they can survive in solutions containing 200 μM arsenate. After 35 days under 100 μM arsenate, transgenic plants displayed lower levels of arsenic in their shoots (28 μg/g dry weight) compared to controls (40 μg/g). Conversely, transgenic roots accumulated more arsenic (2400 μg/g) than wild-type roots (2100 μg/g). Arsenic-free food could be produced by engineering crops with AtACR2 to help reduce soil arsenic levels (Nahar et al. 2017).Using genetically engineered plants (phytoremediation) and genetically modified microorganisms (bioremediation) are two effective and reasonably priced ways to clean up contaminated areas. While bacteria and fungi are among the best at degrading materials, genetically modified plants (GEMs) are better at detoxifying and absorbing metals. These tactics have the ability to address pollution-related problems by utilizing biological processes to degrade pollutants and remove heavy metals from the environment (Arunraja et al. 2023). Figure 5 gives a comparison between conventional and advanced remediation techniques.

#### Physical method

By utilizing the physicochemical characteristics of these metals, physical techniques have been used to remove heavy metals from contaminated systems. Adsorption, membrane filtration, electrokinetic techniques, granular activated carbon, photocatalysis, and soil washing are some of these techniques (Muharrem and Olcay 2017; Akhtar et al. 2020).

Significant attention is given to adsorption processes as they provide a practical method for cleaning up heavy metal-contaminated systems (Khulbe and Matsuura 2018). Notable benefits of this approach include performance, affordability, ease of use, and stability. By using a range of inexpensive adsorbent materials, including biosorbents, clays, activated carbons (Sani et al. 2017), zeolites (Jha and Sivapullaiah 2015), and metal oxides (Akhtar et al. 2020), adsorption technology can effectively lower heavy metal ion concentrations to extremely low levels. Some of the factors influencing the intricate process of metal adsorption onto these materials, particularly agricultural wastes, are complexation, chemisorption, adsorption-complexation on surfaces and pores, micro precipitation, and ion exchange (Akhtar et al. 2020). When biological materials are employed for adsorption, functional groups including sulphydryl, amido, hydroxyl, and carboxyl are crucial for attaching metal ions from water (Khulbe and Matsuura 2018). Numerous studies have demonstrated the effectiveness of different adsorption methods and materials for the removal of heavy metals from contaminated water sources. These approaches offer workable strategies to lessen metal pollution in wastewater; they range from sawdust that is sourced locally to clays that have been biologically changed. Abou-El-Sherbinia and Hassanien (2010) used organically modified montmorillonite clay to extract copper (II) from aqueous solutions. Afkhami et al. (2008) impregnated carbon cloth with ethylenediaminetetraacetic acid (EDTA) to adsorb different metal ions. Ageena (2010) investigated the use of fixed bed adsorption with local sawdust as an adsorbent to remove copper ions from wastewater. Additionally, Amuda et al. (2007) investigated the use of modified activated coconut shell carbon for the removal of heavy metals from industrial effluent.

#### Chemical methods

**Fig. 6:**
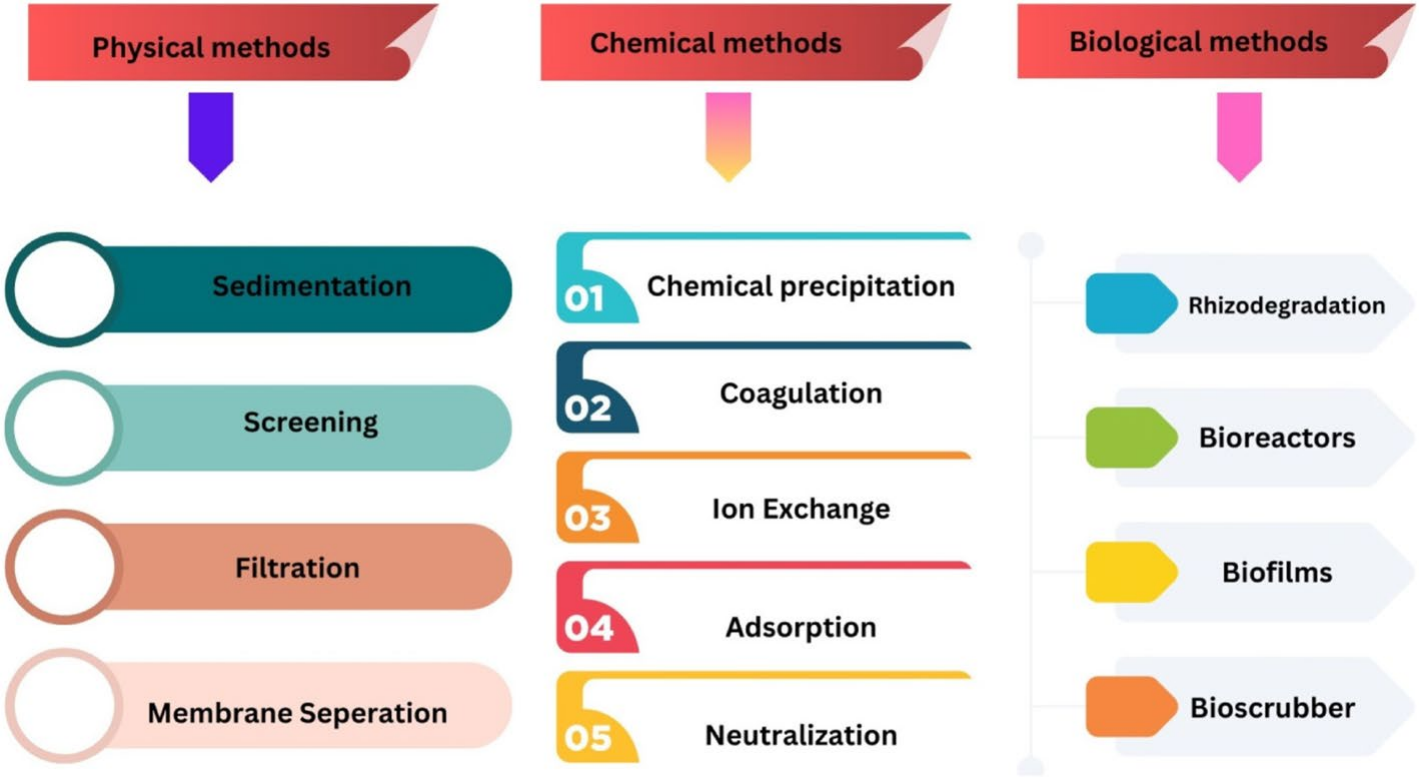
This figure illustrates the various methods employed for the remediation of heavy metals, categorized into physical, i.e. adsorption, filtration. chemical methods i.e. precipitation, ion exchange and biological methods like phytodegradation, bioreactors etc

Chemical processes such as ion exchange, flotation, precipitation, and coagulation/flocculation are crucial for the removal of heavy metals due to their efficacy in water treatment (Akhtar et al. 2020). Insoluble metal hydroxides are produced by adding chemicals during precipitation, and these are then removed from the mixture. The removal of metals by chemical precipitation remains a widely used technique that involves the formation of metal hydroxides with alkaline reagents such as NaOH, Mg (OH)_2_, CaO, and NH_4_OH (Djedidi et al. 2009). Using a two-phase mixture of water and air, flotation techniques in wastewater treatment efficiently remove metal ions by generating aggregates that can be isolated from the solution by using bubbles adhering to the metal ions. Using this method, impurities may be effectively removed from the water and valuable metals such as gold, silver, and palladium are selectively recovered. The flotation technique is a potential method for treating wastewater streams containing metals because it provides thicker concentrations, low operating costs, and good selectivity (Rubio 2002). Ion exchange uses resin materials with specific functional groups to replace metal ions in solution with ions on the resin surface (Lee et al. 2007). Adding coagulants to destabilize colloidal particles and enable them to coalesce into bigger flocs that settle or float for removal is known as flocculation or coagulation (Renault et al. 2009). Excessive use of chemicals, although useful, can have negative effects on the environment and waste management (Renu and Kailash 2017). Figure 6 illustrates these methods used for heavy metal remediation.

Acidic, metal-rich leachates have been treated through precipitation with various chemicals, showing that Ca(OH)_2_ is a more efficient metal remover than NaOH. Additionally, vacuum filtration has a higher dewatering efficiency than centrifugation, and the specific resistance to filtration (SRF) values vary depending on the precipitate (Djedidi et al. 2009). A viable technique for eliminating heavy metals from sludge and industrial wastewaters is ion exchange. Cu^2+^ > Zn^2+^ > Cd^2+^ > H^+^ is the affinity sequence to IR- 120, according to the ion-exchange equilibrium and kinetics employing Amberlite IR- 120 for Cu^2+^, Zn^2+^, and Cd^2+^ systems investigation. The dynamics of the extraction process are analyzed through thermodynamic parameters and ion-exchange kinetics using various models. It is discovered that the activation energies of Cu, Zn, and Cd are 15.41, 7.04, and 17.01 kJ/mol, respectively. According to Lee et al. (2007), the reversible reaction model sheds light on how operating circumstances affect ion-exchange kinetics. As a result, the results can help reduce the amount of metal pollution in industrial effluents by directing the creation of efficient plans for removing heavy metals using ion exchange technology.

## Conclusion and future perspective

Even at low concentrations, heavy metal contamination has been shown to have detrimental impacts on human health and environmental ecosystems. It is essential to comprehend how plants react to heavy metal stress in order to develop efficient mitigation techniques. Phytoremediation, which involves the use of plants and associated microbes, presents a viable method for removing heavy metals from contaminated soils. Advances in biotechnology, such as omics technologies and genetic engineering, have the potential to increase plant resistance to heavy metal toxicity. To mitigate heavy metal pollution and promote environmental health, comprehensive and sustainable solutions must be developed through collaborative efforts. Additional investigation is required to enhance our comprehension of the molecular reactions of plants to heavy metal stress and to investigate cooperative strategies that integrate biological, biotechnological, and physicochemical techniques. The creation of novel interventions can be informed by complete insights obtained from the integration of multi-omics methods. We can greatly address the problems caused by heavy metal contamination and move toward a healthier and more robust future by utilizing scientific breakthroughs.

## Data Availability

There is no additional associated data with this article.

## Abbrevations

As: Arsenic
Ca^2+^: Calcium
Cd: Cadmium
Cr: Chromium
Cu: Copper
DMA: Dimethylarsinic acid
DNA: Deoxyribonucleic acid
EDTA: Ethylenediaminetetraacetic acid
ET: Ethylene
Fe: Iron
FIT: FER-like Deficiency Induced Transcription Factor
GC-MS: Gas chromatography coupled with mass spectrometry
GEMs: Genetically modified plants
GSH: Glutathione
GST: Glutathione S-transferase
H_2_O_2_: Hydrogen peroxide
HMs: Heavy Metals
HPLC: High-performance liquid chromatography
ICP-MS: Inductively coupled mass spectrometry
JA: Jasmonic acid
LC-MS: Liquid chromatography coupled with mass spectrometry
LHCII: Light-harvesting complex II
MAPK: Mitogen-activated protein kinase
Mg: Magnesium
Mn: Manganese
MMA: Monomethylarsinic acid
MTs: Metallothioneins
MYB: MYB transcription factors
MYC: MYC transcription factors
NAC: NAC transcription factors
Ni: Nickel
NMR: Nuclear magnetic resonance spectroscopy
OH^•^: Hydroxyl radical
O_2_–: Superoxide anion
Pb: Lead
PCs: Phytochelatins
Pi: Inorganic phosphate
ROS: Reactive oxygen species
SA: Salicylic acid
SDMs: Significantly different metabolites
Se: Selenium
SOD: Superoxide dismutase
SRF: Specific resistance to filtration
TFs: Transcription factors
UHPLC/LC-MS: Ultra-high performance liquid chromatography coupled with mass spectrometry
WRKY: WRKY transcription factors
Zn: Zinc
